# Combined immune checkpoint inhibition with durvalumab and tremelimumab with and without radiofrequency ablation in patients with advanced biliary tract carcinoma

**DOI:** 10.1002/cam4.6912

**Published:** 2024-01-11

**Authors:** Cecilia Monge, Changqing Xie, Yuta Myojin, Kelley L. Coffman‐D'Annibale, Donna Hrones, Gagandeep Brar, Sophie Wang, Anuradha Budhu, William D. Figg, Maggie Cam, Richard Finney, Elliot B. Levy, David E. Kleiner, Seth M. Steinberg, Xin Wei Wang, Bernadette Redd, Bradford J. Wood, Tim F. Greten

**Affiliations:** ^1^ Gastrointestinal Malignancies Section, Thoracic and GI Malignancies Branch, Center for Cancer Research, National Cancer Institute National Institutes of Health Bethesda Maryland USA; ^2^ Laboratory of Human Carcinogenesis, Center for Cancer Research, National Cancer Institute National Institutes of Health Bethesda Maryland USA; ^3^ Liver Cancer Program, Center for Cancer Research, National Cancer Institute National Institutes of Health Bethesda Maryland USA; ^4^ Genitourinary Malignancies Branch, Center for Cancer Research, National Cancer Institute National Institutes of Health Bethesda Maryland USA; ^5^ Center for Collaborative Bioinformatics, Center for Cancer Research, National Cancer Institute National Institutes of Health Bethesda Maryland USA; ^6^ Center for Interventional Oncology, Center for Cancer Research, National Cancer Institute National Institutes of Health Bethesda Maryland USA; ^7^ Laboratory of Pathology, Center for Cancer Research, National Cancer Institute National Institutes of Health Bethesda Maryland USA; ^8^ Biostatistics and Data Management Section, Center for Cancer Research, National Cancer Institute National Institutes of Health Bethesda Maryland USA; ^9^ Radiology and Imaging Sciences, Center for Cancer Research, National Cancer Institute National Institutes of Health Bethesda Maryland USA

**Keywords:** biliary tract cancer, cholangiocarcinoma, cryoablation, durvalumab, immune checkpoint inhibitor, interventional radiology, radiofrequency ablation, tremelimumab

## Abstract

**Background:**

Current standard of care for advanced biliary tract cancer (BTC) is gemcitabine, cisplatin plus anti‐PD1/PD‐L1, but response rates are modest. The purpose of this study was to explore the efficacy and safety of durvalumab (anti‐PD‐L1) and tremelimumab (anti‐CTLA‐4), with and without an interventional radiology (IR) procedure in advanced BTC.

**Methods:**

Eligible patients with advanced BTC who had received or refused at least one prior line of systemic therapy were treated with tremelimumab and durvalumab for four combined doses followed by monthly durvalumab alone with and without an IR procedure until the progression of disease or unacceptable toxicity. Objective response was assessed through CT or MRI by Response Evaluation Criteria in Solid Tumors (RECIST, version 1.1) every 8 weeks. Adverse events (AEs) were recorded and managed. The primary endpoint was 6‐month progression‐free survival (PFS).

**Results:**

Twenty‐three patients with advanced BTC were enrolled; 17 patients were assigned to treatment with durvalumab and tremelimumab (Durva/Treme); and 6 patients were treated with the combination of durvalumab, tremelimumab plus IR procedure (Durva/Treme + IR). The best clinical responses in the Durva/Treme arm were partial response (*n* = 1), stable disease (*n* = 5), progressive disease (*n* = 5), and in the Durva/Treme + IR arm: partial response (*n* = 0), stable disease (*n* = 3), progressive disease (*n* = 3). The median PFS was 2.2 months (95% CI: 1.3–3.1 months) in the Durva/Treme arm and 2.9 months (95% CI: 1.9–4.7 months) in the Durva/Treme + IR arm (*p* = 0.27). The median OS was 5.1 months (95% CI: 2.5–6.9 months) in the Durva/Treme arm and 5.8 months (95% CI: 2.9–40.1 months) in the Durva/Treme + IR arm (*p* = 0.31). The majority of AEs were grades 1–2.

**Conclusion:**

Durva/Treme and Durva/Treme + IR showed similar efficacy. With a manageable safety profile. Larger studies are needed to fully characterize the efficacy of Durva/Treme ± IR in advanced BTC.

## Graphical abstract

The purpose of this study was to explore the efficacy and safety of two ICIs, durvalumab and tremelimumab, with and without an interventional radiology (IR) procedure in patients with advanced BTC. Durva/Treme + IR showed no difference in efficacy compared with Durva/Treme alone, but the safety profile of both cohorts was manageable. Larger studies are needed to fully characterize efficacy of Durva/Treme with or without IR in advanced BTCs.

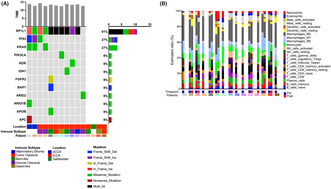

## 1 INTRODUCTION

Biliary tract cancer (BTC) is a rare but aggressive malignancy that arises from the epithelial cells lining the bile ducts, gallbladder, and liver.^1^, ^2^ Surgical resection or liver transplantation can be curative for patients with early stage disease, however, the majority of patients either recur or initially present with advanced disease.^3^, ^4^ The prognosis for BTC in the metastatic setting is generally poor, with a 5‐year relative survival rate of only 2%–5%.^5^, ^6^ This low survival rate is partly due to the lack of effective treatment options for patients with BTC but with recent advancements in both the understanding of the biology of BTC and technologies such as next‐generation gene‐sequencing, novel potential treatments for advanced BTC continue to emerge.

ICIs, targeting cytotoxic T‐lymphocyte‐associated protein 4 (CTLA‐4) and programmed death ligand‐1 (PD‐1/PD‐L1), pathways have revolutionized the treatment of several solid tumors.^7^ Like other solid tumors, BTC elicits and evades effective immune responses.^8^ Inhibiting immune checkpoint proteins gives the cell the opportunity to elicit a strong immune response and allows T cells to kill cancer cells. Monotherapy with an ICI has shown limited overall response rates (ORR) in BTC,^9^, ^10^, ^11^, ^12^ but ICI in combination with systemic therapy such as combination chemotherapy with gemcitabine and cisplatin has been shown to improve overall survival (OS).^13^, ^14^ Although this combination chemotherapy + ICI is now the standard of care in the first‐line setting,^15^, ^16^ the response rates are still modest, and the duration of response is often short‐lived.^8^, ^17^

Tumor mutation burden (TMB), the total number of noninherited mutations per million base of DNA, is used as a predictive biomarker for response to immunotherapy in patients. In general, the higher the TMB, the greater the difference between the tumor tissue versus normal tissue, and the better the response to immunotherapy.^18^ For patients who progress on first‐line therapy, precision medicine can be employed based on tumor genomics. It is estimated that ~40% of patients with BTC harbor at least one targetable oncogenic genetic alteration including changes in *IDH1/2*, *FGFR2*, *KRAS*, *BRAF*, *ERBB2*, *NTRK*, *ROS*, *RET*, and other genes encoding mismatch repair enzymes.^19^ Some selective inhibitors of such oncogenic drivers have been shown to improve progression‐free survival (PFS), OS, and ORR, however extensive differences in response to precision medicine treatment regimens across different BTC subtypes have been reported and acquired resistance is nearly ubiquitous.^20^, ^21^, ^22^, ^23^, ^24^, ^25^, ^26^ In patients without targetable oncogenic mutations, treatment with fluoropyridine chemotherapy combinations, compounds that work by interfering with DNA synthesis, leads to modest improvement.^27^, ^28^

Interventional radiology (IR) techniques like cryoablation (CRYO) and radiofrequency ablation (RFA) are emerging as promising local therapies for the treatment of BTC. Several studies have reported the efficacy of these techniques in controlling local disease and improving survival outcomes for patients with advanced BTC.^29^, ^30^, ^31^ CRYO and RFA have been shown to be safe and effective in relieving biliary obstruction and we and others have shown that they can be used in combination with other therapies such as chemotherapy or immunotherapy to achieve better outcomes for some patients.^10^ Further studies are needed to explore the full potential of these combined therapies in the management of BTC.

We initially tested tremelimumab in combination with microwave ablation in refractory BTC.^10^ This trial was initiated based on positive results which we had observed in patients with hepatocellular carcinoma treated with tremelimumab plus locoregional therapies.^32^ The purpose of this study was to study the safety and efficacy of the combination of tremelimumab plus durvalumab in patients with advanced BTC and to determine if the addition of IR to combined ICI therapy (tremelimumab and durvalumab) could improve outcomes for patients with advanced BTC since tremelimumab monotherapy had minimal effects in BTC.^10^ The study design included two cohorts of patients with advanced BTC who had progressed on first‐line therapy. Cohort 1 received durvalumab and tremelimumab, whereas Cohort 2 received the same combination together with an IR procedure. The primary endpoint was PFS. OS, ORR, and safety were also analyzed.

## 2 MATERIALS AND METHODS

### 2.1 Patients

Eligible patients were at least 18 years old and had histopathological confirmation of BTC by the Laboratory of Pathology at the National Cancer Institute (NCI). Patients must have had a disease that was not amenable to potentially curative resection, transplantation, or ablation. Patients must have received, been intolerant of, or refused at least one line of chemotherapy in the advanced setting. The required ECOG performance status was 0–2 and normal organ and marrow function were inclusion criteria. A history of chronic autoimmunity or inflammatory bowel disease was considered exclusion criteria. All patients provided written informed consent and the study was approved by the NCI Institutional Review Board. All cases were discussed at an NCI multidisciplinary gastrointestinal tumor board. The ClinicalTrials.gov identifier was NCT02821754.

### 2.2 Study design

Patients who met the eligibility criteria were enrolled in the study. All patients received durvalumab 1500 mg intravenously (IV) and tremelimumab 75 mg IV every 4 weeks for 4 cycles followed by monthly durvalumab 1500 mg IV alone every 4 weeks until disease progression or intolerance. Patients in Cohort 1 received only the drug regimen. Patients in Cohort 2 received the drug regimen and additionally underwent an IR procedure on day 36. Imaging studies were performed by contrast‐enhanced CT or MRI scan every 8 weeks. Objective response was assessed by Response Evaluation Criteria in Solid Tumors (RECIST, version 1.1). Tumor biopsies were performed at baseline; before the first ICI administration and at day 36 (postsecond ICI administration). The primary objective was to evaluate the 6‐month PFS of combining tremelimumab and durvalumab with or without IR in patients with advanced BTC. The secondary objective was to assess the safety and feasibility of combining tremelimumab and durvalumab with and without IR in patients with BTC. Microwave ablation was only performed in those patients where it was felt to be beneficial for the patients based on limited tumor size. We also looked at change in tumor size. Safety and toxicity were monitored and managed accordingly.

### 2.3 Safety and efficacy

All adverse events (AEs) and serious AEs occurring within 30 days of the last dose were reported according to the NCI Common Terminology Criteria for Adverse Events v 4.0. All patients were evaluable for toxicity from the time of their first treatment with tremelimumab and durvalumab. The evaluable population was defined as all patients who had received at least one cycle of therapy and had their disease re‐evaluated on imaging using RECIST 1.1. In addition, PFS, and OS were calculated by the Kaplan–Meier method and reported as medians, ratios, and 95% CIs.

### 2.4 Gene expression analysis

Baseline biopsies and on‐treatment biopsy samples were obtained. Tumor samples were stored as fresh‐frozen at deep freeze. DNA and RNA were extracted, sequenced, and analyzed as previously described. Whole‐exon sequence was performed to analyze specific mutations of samples and TMB. RNA‐seq analysis was performed to examine gene expression. For immune subtype classification, the enrichment score for each class of baseline samples was calculated with GSVA analysis using a published gene list.^33^ CIBERSORTx as a deconvolution method was used to align samples according to the immune subtypes. The TPM gene expression matrix output by RSEM was used and LM22 was used as a gene signature matrix, as previously described.^34^, ^35^

#### 2.4.1 Bulk RNA‐seq and data analysis

RNA was extracted from fresh‐frozen needle biopsy tumor samples at baseline and post‐treatment as previously described.^36^ Libraries were prepared using the NEBNext® Ultra™ II Directional RNA Library Prep Kit for Illumina, using the 100 or 5 ng protocols based on RNA availability at the CCR Sequencing Facility. Samples with <5 ng RNA (“low concentration”) were prepared using the 5 ng protocol. Sequencing data were generated by the CCR Sequencing Facility. Ninety‐nine samples were sequenced on a NovaSeq S2 flowcell (2 × 151 bp); 72 with good yield were then sequenced in greater depth on a NovaSeq S4 flowcell (2 × 151 bp), and reads were combined. We used the Pangea RNA‐Seq Pipeline (https://github.com/NCIPangea/RNA‐seek) to process the data. Briefly, sequences in fastq format were trimmed using bbtools version 38.87.^37^ The trimmed sequences were aligned using STAR 2.7.6a using the gencode.v36.annotation.gtf and hg38 Ref. [38]. RSEM version 1.3.0 was used to quantify the aligned data to produce a two‐dimensional (genes × samples) gene counts matrix.^39^

#### 2.4.2 Tumor profiling analysis

Downstream analysis and visualization were performed within the NIH Integrated Analysis Platform (NIDAP) using R programs developed on the Foundry platform (Palantir Technologies). The gene counts matrix was imported into the NIDAP platform, where genes were filtered for low counts (<1 cpm) and normalized by quantile normalization using the limma package.^40^ The normalized data was further analyzed using Gene Set Variation Analysis (GSVA).^41^ Furthermore, CIBERSORTx was performed to estimate the proportion of immune cells in a bulk sample as previously described.^36^ The TPM gene expression matrix output by RSEM was reformatted to include only gene symbols. Immune infiltration was profiled using CIBERSORTx^34^ with the LM22 signature matrix in absolute mode with batch correction. The relative abundance of each cell type was calculated as a proportion of the absolute score. Statistical analysis of the CIBERSORT immune cell type proportions showed no significance among all the different cell types between treated and control samples. All but one primary liver sample had a *p*‐value <0.05 (*n* = 200).

#### 2.4.3 Statistical analysis

The trial was originally designed to enroll up to 7 individual small cohorts with up to 10 patients enrolled in each, for a pilot evaluation distinguished by disease type and treatment, aiming to see if historical proportions stable of 10%–15% could be improved upon. However, for simplicity, and based on the actual observed types of patients enrolled, the cohorts in this manuscript only refer to the distinction between the broad classes of treatments administered, with multiple disease types combined in this evaluation. Statistical analysis was performed using SAS version 9.4 and GraphPad Prism9 software. OS was calculated from the on‐study date until the date of death. PFS was calculated from the on‐study date until the date of progression, or the date of death if a patient who remained on study died prior to progression. The probabilities of OS and PFS were calculated by the Kaplan–Meier method, with the statistical significance of the difference between two Kaplan–Meier curves determined by the log‐rank test.

## 3 RESULTS

### 3.1 Patient characteristics

**FIGURE 1:**
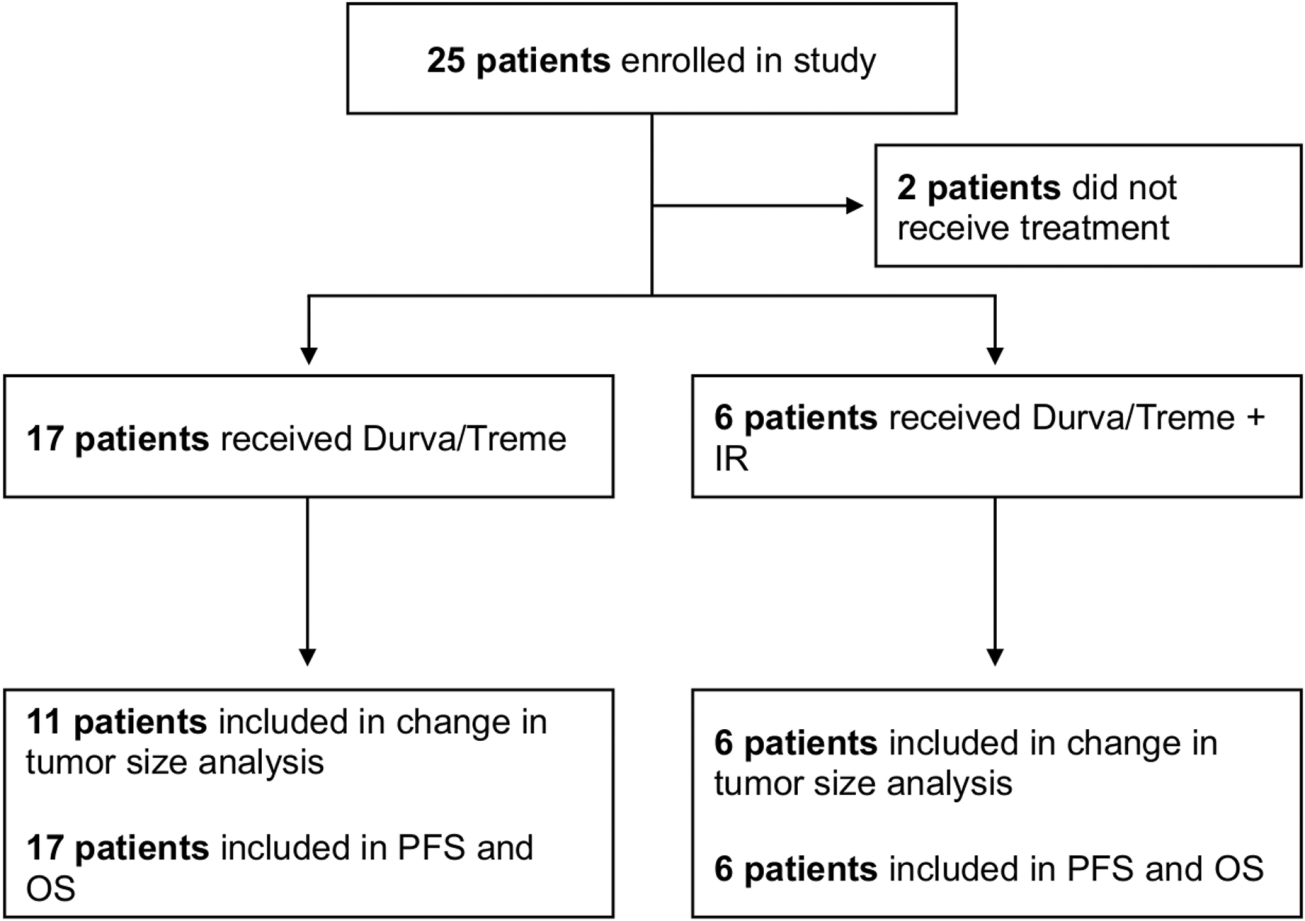
Study enrollment profile.

Twenty‐five patients with BTC were enrolled in this study including 20 patients with intrahepatic cholangiocarcinoma (iCCA), one patient with extrahepatic cholangiocarcinoma (eCCA), and two patients with gallbladder cancer. None of the patients had received prior treatment with immune checkpoint inhibitors. After registration, two patients opted to not continue on the trial and never received treatment. The median age of the remaining 23 patients was 63 years (range: 21–80). Patients were registered to one of two cohorts. Cohort 1 received durvalumab and tremelimumab (Durva/Treme) only and Cohort 2 received Durva/Treme and underwent an IR procedure (Durva/Treme + IR) (Figure 1). Extrahepatic metastases were present in 82% of patients in Cohort 1 and 100% of patients in Cohort 2. One hundred percent of patients in both cohorts had received previous treatment including chemotherapy (100% in both cohorts), radiotherapy (29% in Cohort 1 and 33% in Cohort 2), and/or surgical resection (41% in Cohort 1 and 83% in Cohort 2). All baseline characteristics for patients in both cohorts are shown in Table 1.

**TABLE 1:** Baseline characteristics.

	Cohort 1	Cohort 2
	Durva/Treme (*n* = 17)	Durva/Treme + IR (*n* = 6)
Age in years, median^42^	63 (21–80)	64 (53–69)
Sex, no. (%)		
Female	7 (41%)	1 (17%)
Male	10 (59%)	5 (83%)
Race		
White, no. (%)	16 (94%)	6 (100%)
Asian, no. (%)	1 (6%)	‐
African American, no. (%)	‐	‐
Other/Unknown, no. (%)	‐	‐
ECOG performance status		
0, no. (%)	7 (41%)	2 (33%)
1, no. (%)	10 (59%)	4 (67%)
Baseline values (K/μL), median		
abs Lympho	1.11	1.36
CA19‐9 (U/mL)	145.9	83.3
Longest tumor diameter (cm), median	6.9	4
Positive for extrahepatic metastases, no. (%)	14 (82%)	6 (100%)
Number of lesions		
1, no. (%)	1 (6%)	‐
2, no. (%)	1 (6%)	‐
3–5, no. (%)	8 (47%)	‐
>5, no. (%)	7 (41%)	6 (100%)
Primary tumor site, no. (%)		
Intrahepatic	15 (88%)	5 (83%)
Extrahepatic	1 (6%)	‐
Gallbladder	1 (6%)	1 (17%)
Previous treatment, no. (%)	17 (100%)	6 (100%)
Surgical resection	7 (41%)	5 (83%)
Radiotherapy	5 (29%)	2 (33%)
Chemotherapy	17 (100%)	6 (100%)
1 line, no. (%)	6 (35%)	1 (17%)
2 lines, no. (%)	7 (41%)	2 (33%)
≥3 lines, no. (%)	4 (24%)	1 (17%)

**FIGURE 2:**
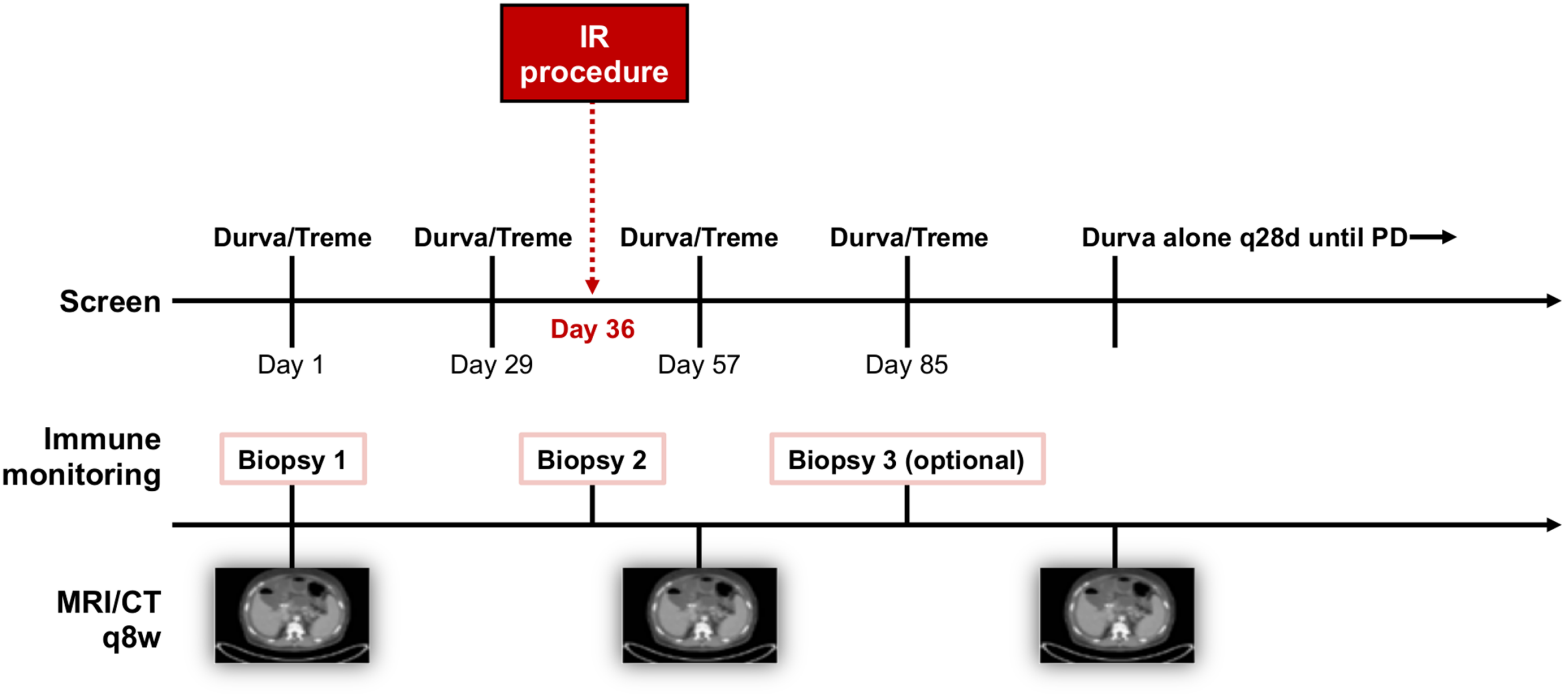
Treatment paradigm. Patients were treated with durvalumab 1500 mg intravenously (IV) and tremelimumab 75 mg (IV) every 4 weeks for 4 cycles followed by monthly durvalumab 1500 mg IV alone until progression of disease or unacceptable toxicity. Patients in Cohort 1 received drug only. Patients in Cohort 2 received drug and additionally underwent an interventional radiology (IR) procedure on day 36. All patients received a baseline biopsy and on‐treatment biopsy at the time of the interventional radiation procedure. An optional third biopsy was requested on day 85. MRI or CT scan performed every 8 weeks.

Patients in Cohort 1 (*n* = 17) were treated with durvalumab 1500 mg intravenously (IV) and tremelimumab 75 mg IV every 4 weeks for four cycles followed by monthly durvalumab 1500 mg IV alone until progression of disease or unacceptable toxicity. Patients in Cohort 2 (*n* = 6) were treated with the same regimen as Cohort 1 and additionally underwent an IR procedure on day 36 (Figure 2).

### 3.2 Efficacy

**FIGURE 3:**
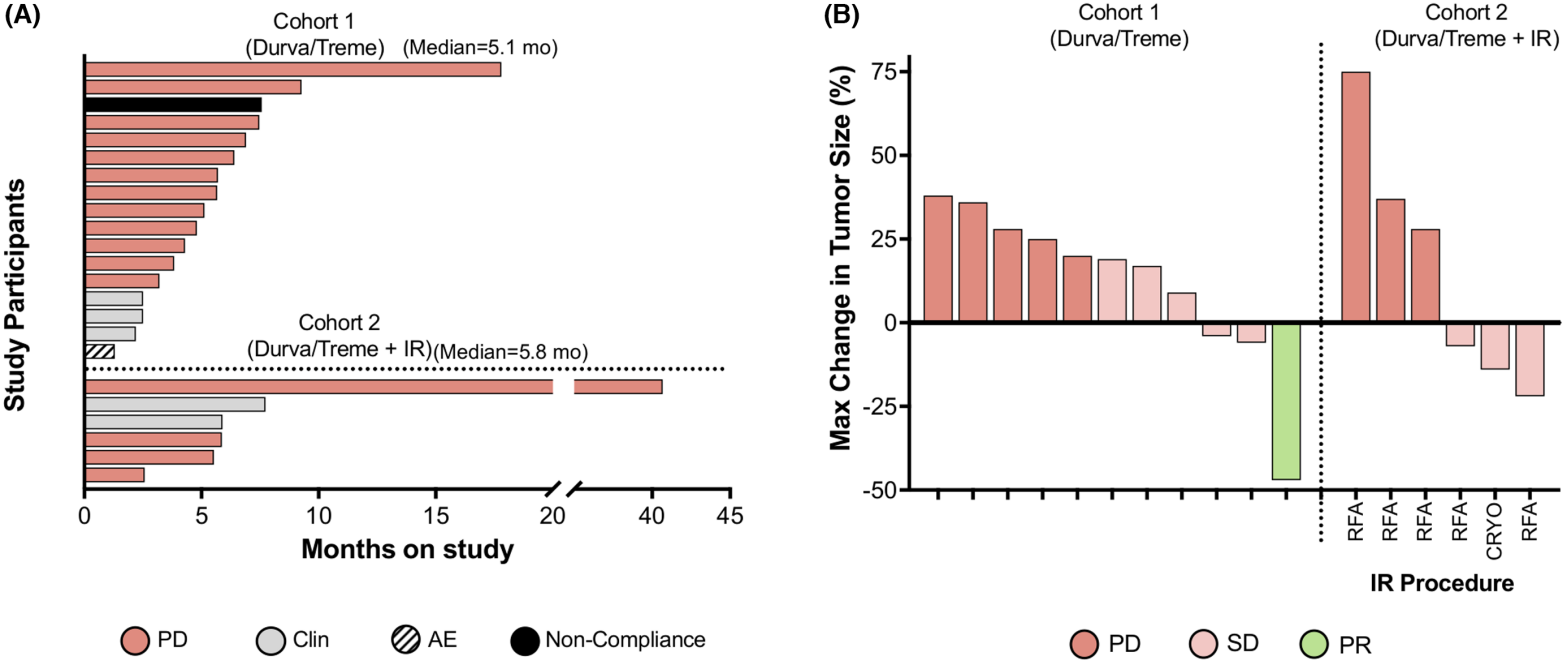
Efficacy results. (A) Number of months between the on‐study date and the date of death for participants in Cohort 1 and Cohort 2. Colors in (A) represent the reason for treatment termination. (B) Maximal change in tumor size in participants in Cohort 1 and Cohort 2. Colors in (B) represent a clinical response to treatment. AE, adverse event; clin, clinical; PD, progressive disease; PR, partial response; SD, stable disease.

The median follow‐up on the study was 5.1 months for Cohort 1 and 5.8 months for Cohort 2 (Figure 3A). Eleven patients in Cohort 1 and six patients in Cohort 2 had lesions that were evaluable for response measured by change in tumor size. Between both cohorts, there was one partial response giving an ORR of 4%. The partial response lasted for approximately 4 months. Eight patients had stable disease for a disease control rate of 30%. Eight patients continued to have progressive disease after treatment (Table 2; Figure 3B).

**TABLE 2:** Treatment response.

	Cohort 1	Cohort 2	Cohort 1 + 2 (*n* = 23)
	Drug only (*n* = 17)	Drug + IR (*n* = 6)	
Clinical response			
PD, no. (%)	5 (29%)	3 (50%)	8 (34.8%)
SD, no. (%)	5 (29.5%)	3 (50%)	8 (35%)
PR, no. (%)	1 (6%)	‐	1 (4%)
Unevaluable, no. (%)	6 (35.3%)	‐	6 (26%)
Survival			
Median PFS, months (95% CI)	2.2 (1.3–3.1)	2.9 (1.9–4.7)	2.6
Median OS, months (95% CI)	5.1 (2.6–6.9)	5.8 (2.9–40.1)	5.6

**FIGURE 4:**
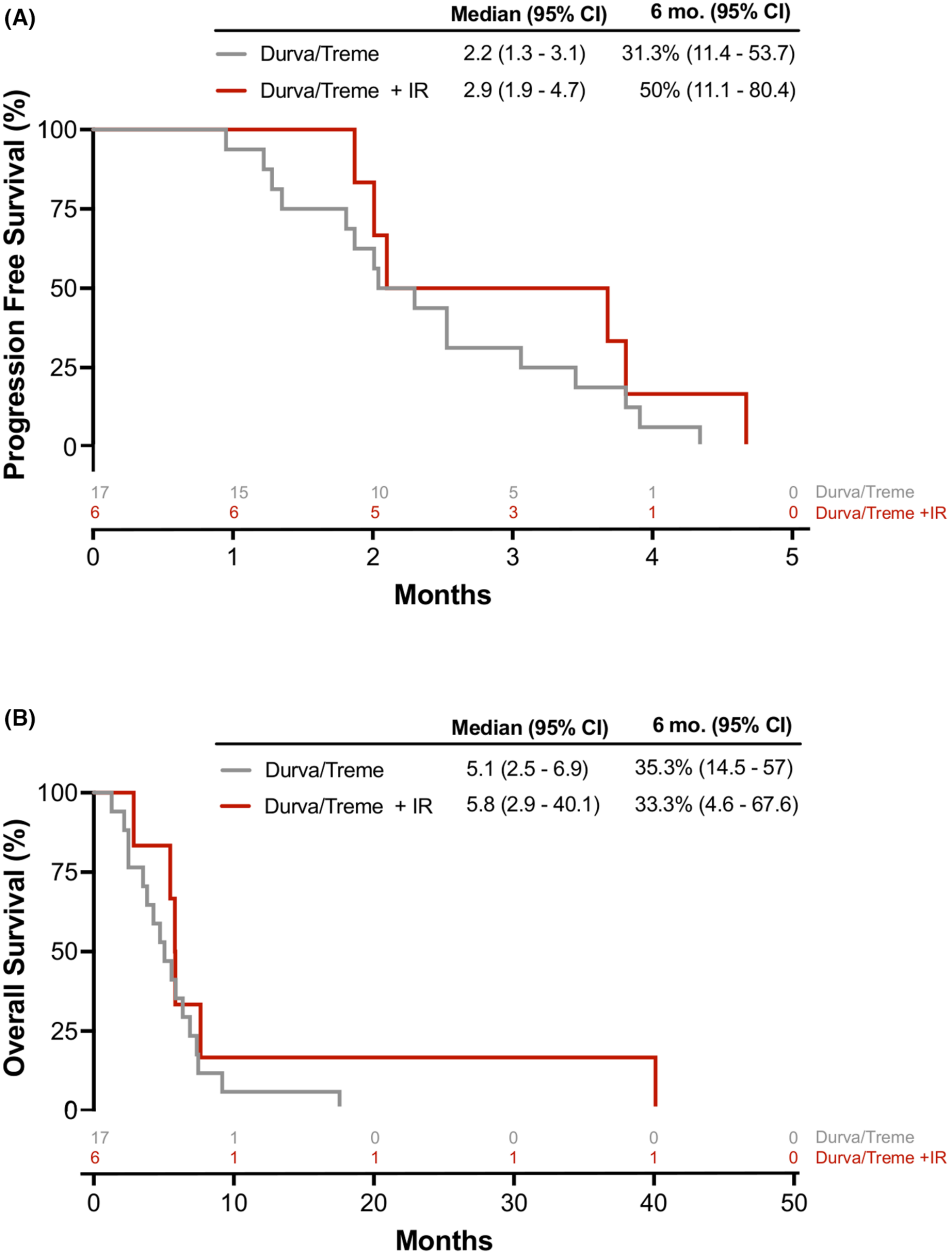
Survival plots. (A) Progression‐free survival (PFS) and (B) overall survival (OS) for participants in Cohort 1 and Cohort 2. No significant difference between the two cohorts for PFS (0.27) or OS (*p* = 0.31).

Both PFS and OS were calculated for 17 patients in Cohort 1 and six patients in Cohort 2. Median PFS was 2.2 months (95% CI: 1.3–3.1 months) for Cohort 1, 2.9 months (95% CI: 1.9–4.7 months) for Cohort 2 (*p* = 0.27), and 2.2 months (95% CI: 1.9–3.4 months) for both cohorts combined (Figure 4A; Figure S1A; Table 2). All fully treated patients progressed within 6 months; one patient who received one dose of treatment was censored in the curves for PFS after 3 weeks because of noncompliance; the patient was included because a minimal amount of treatment was given. Median OS was 5.1 months (95% CI: 2.5–6.9 months) for Cohort 1, 5.8 months (95% CI: 2.9–40.1 months) for Cohort 2 (*p* = 0.31), and 5.6 months (95% CI: 3.8–6.3 months) for both cohorts combined (Figure 4B; Figure S1B; Table 2). No disease‐specific subset analyses were performed given the small number of patients.

### 3.3 Safety

A total of 113 treatment‐related AEs were reported (Table 3). All patients experienced at least one treatment‐related AE, with the majority being grade 1–2 (58%) where medical intervention was not required. Grade 3 AEs were seen in 52% of patients, with the majority being a laboratory finding including lymphopenia, increase in ALT, Alk Phos, or AST. There were no grade 4 AEs and one grade 5 AE; myasthenia gravis (which was resolved with treatment consisting of steroids and immunoglobulins). The most commonly occurring treatment‐related AEs were lymphopenia, AST increase, pruritus, fatigue, and anemia. Eight patients experienced an immune‐related AE; there were no deaths secondary to AE's.

**TABLE 3:** Treatment‐related adverse events (AEs).

Adverse events	Cohort 1			Cohort 2		
	Durva/Treme (*n* = 17)			Durva/Treme +IR (*n* = 6)		
	No. patients (%)			No. patients (%)		
	Grade 1–2	Grade 3	Grade 5	Grade 1–2	Grade 3	Grade 5
Alk Phos increase	3	2	‐	‐	‐	‐
ALT increase	3	2	‐	‐	‐	‐
Amylase increase	‐	‐	‐	1 (17%)	‐	‐
Anemia	5	‐	‐	3	‐	‐
Arthralgia	‐	‐	‐	1 (17%)	1 (17%)	‐
Anorexia	1	‐	‐	‐	‐	‐
AST increase	7	3	‐	1 (17%)	‐	‐
Bilirubin increase	1	‐	‐	‐	‐	‐
Blurred vision	‐	‐	‐	1 (17%)	‐	‐
Cough	‐	‐	‐	1 (17%)	‐	‐
Dehydration	1	‐	‐	‐	‐	‐
Diarrhea	1	‐	‐	‐	‐	‐
Dry skin	1	‐	‐	‐	‐	‐
Fatigue	6	2	‐	2	‐	‐
Fever	1	1	‐	‐	‐	‐
Flank pain	‐	‐	‐	‐	‐	‐
Hypercalcemia	1	‐	‐	‐	‐	‐
Hyponatremia	2	‐	‐	‐	‐	‐
Hypophosphatemia	2	1	‐	1 (17%)	‐	‐
Insomnia	‐	‐	‐	1 (17%)	‐	‐
Itching/pruritus	6	1	‐	2	‐	‐
Low WBC	2	‐	‐	3	‐	‐
Lymphopenia	13	1	‐	6	2	‐
Malaise	‐	‐	‐	1 (17%)	‐	‐
Myalgia	‐	‐	‐	2	1 (17%)	‐
Platelet count decreased	‐	‐	‐	2	‐	‐
Supraventricular tachycardia	‐	1	‐	‐	‐	‐
Weight loss	1	‐	‐	‐	‐	‐
Immune‐related AEs						
Autoimmune Hepatitis	2	‐	‐	‐	‐	‐
Hypothyroiditis	‐	‐	‐	2	‐	‐
Rash	2	1	‐	1 (17%)	‐	‐
Myositis	‐	‐	‐	‐	1 (17%)	‐
Myastenia gravis	‐	‐	1	‐	‐	‐

### 3.4 Correlative studies

**FIGURE 5:**
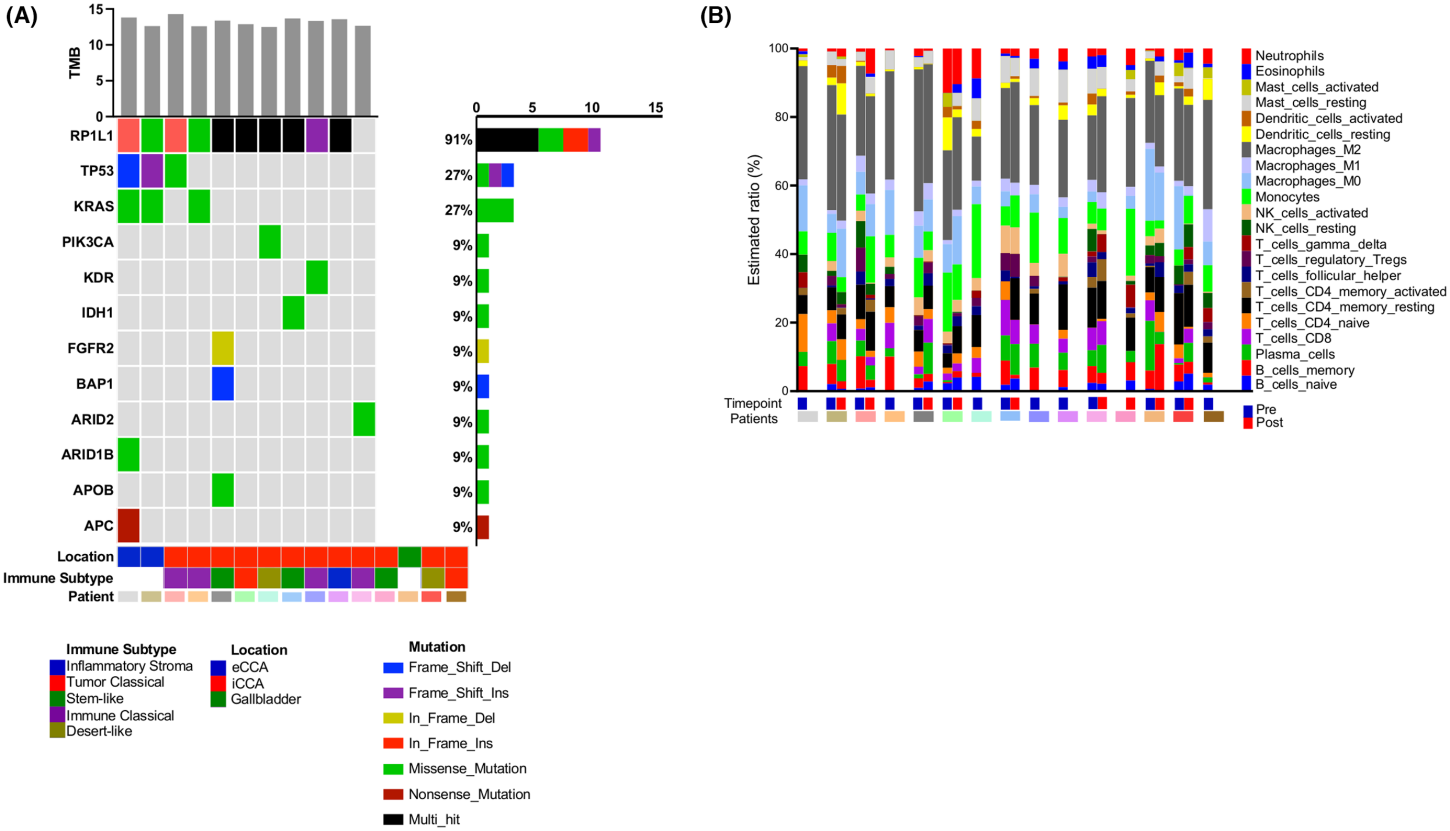
Gene expression and somatic variant analysis of tumor biopsies. (A) Oncoplot of known driver genes in baseline tumor samples; gene expression (RNASeq) for CIBERSORTx. The top bar plot presents the tumor mutation burden (TMB) from WES per sample, defined as the number of nonsynonymous mutations per Mb of coding region. The right bar plot presents the number and percent of samples in which the gene is mutated. Samples were analyzed for immune cell subsets according to Job et al.^35^ (B) Relative abundance of different immune cell types in baseline tumors, as estimated by CIBERSORTx.

We explored TMB and composition of immune cells in biopsies obtained at baseline and on treatment. Specific mutations are shown in Figure 5A. The TMB of the biopsies was below 15 nonsynonymous mutations per Mb of coding region for all samples, a relatively low TMB (Figure 5A). Composition of immune cells was analyzed in biopsies obtained at baseline and on‐treatment. A clear increase in CD8^+^ T cells was seen only in one (out of eight total) biopsies and this patient progressed upon immune checkpoint inhibitor therapy (Figure 5B). No other clear changes upon immune checkpoint inhibitor therapy were observed.

**FIGURE 6:**
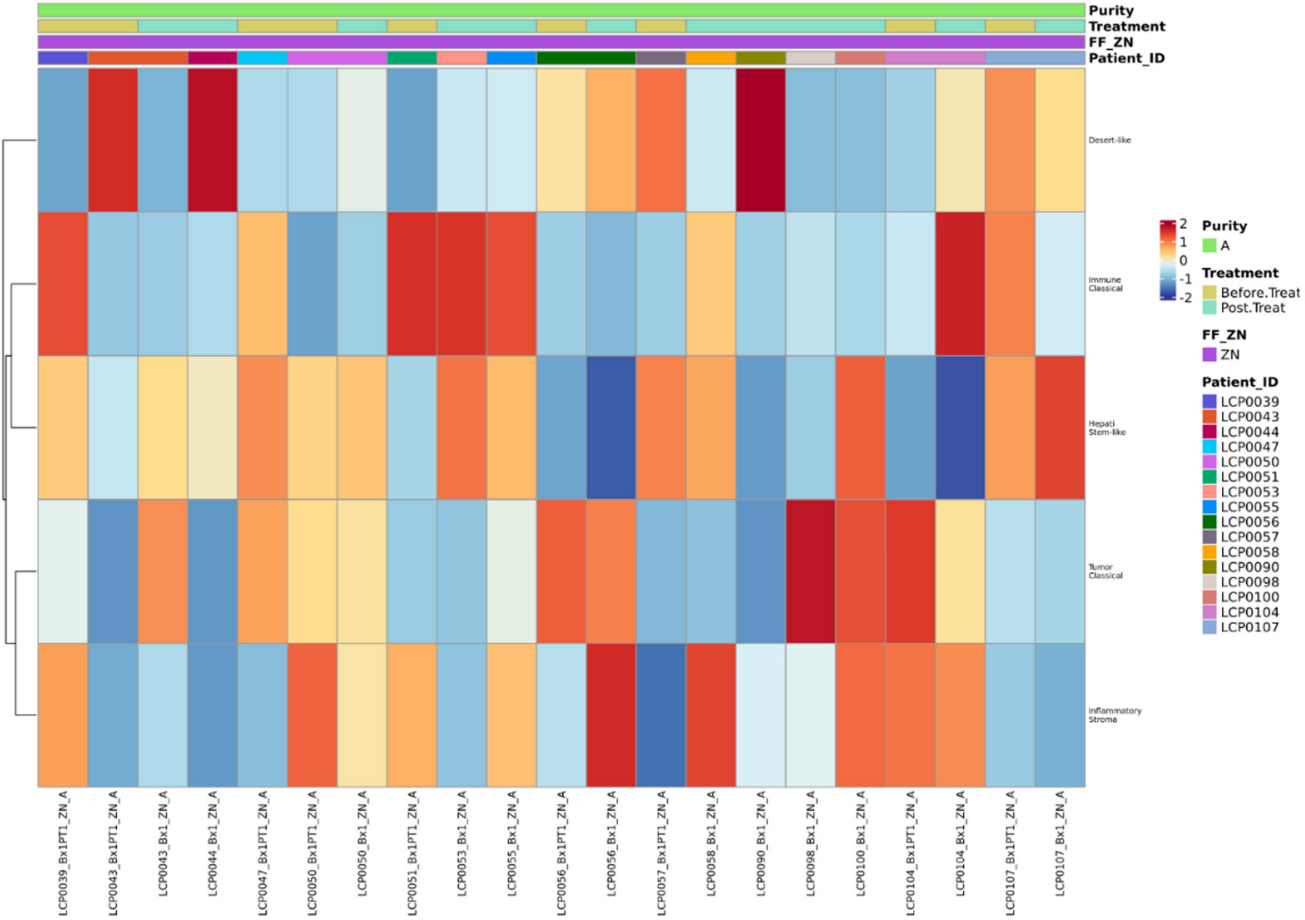
Immune cell profiles of tumor biopsies. RNAseq results from tumor biopsies from 15 patients. Immune cell subtypes are shown on the right of matrix plot. All five previously described immune subtypes were present. The “immune classical” subtype was the most common subtype.

As an exploratory trial endpoint, pharmacodynamic effects and potential underlying immune mechanisms were studied via profiling of tumor biopsies from 15 patients including 8 patients from which tumor samples at baseline and on treatment (day 36) were available. Tumor biopsies underwent bulk RNA‐sequencing analysis. The TMB (nonsilent mutations per Mb of coding area, 35.7 Mb) was not significantly different across all samples tested and mutational profiles were similar to what we had seen in earlier studies (Figure 5). Gene Set Variation Analysis of the gene expression data using the stroma, tumor, and immune microenvironment (STIM) classifier from Martin‐Serrano et al.^33^ revealed that all five previously described immune subtypes of ICC were present (Figure 6). The “immune classical” subtype was the most common subtype (Figure 5 filtered to show only frozen samples) making it the dominant signature in 5 out of 12 patients. Using the CIBERSORTx deconvolution tool, we were able to profile the immune infiltrate, which did not reveal significant differences in the proportion of any immune cell type measured between posttreatment and baseline samples.

## 4 DISCUSSION

This study aimed to evaluate the safety and efficacy of durvalumab and tremelimumab with and without IR in patients with advanced BTC who had previously received at least one line of systemic therapy. This study was based on the hypothesis that combining IR with ICI could selectively enhance antitumor immunity and therefore improve PFS and OS.^43^

The study analyzed 23 patients with advanced BTC, and the primary endpoint was 6‐month PFS. The results showed that both Durva/Treme and Durva/Treme + IR were well tolerated with manageable AEs. Most AEs were grade 1–2 and only one patient had a grade 5 AE; myasthenia gravis, which was resolved with medical management. The safety profiles of both cohorts were similar suggesting that combining IR with ICI does not lead to excess toxicity in patients with advanced BTC.

Historically, monotherapy ICI in combination with microwave ablation demonstrated a median PFS of 3.4 months and an OS of 6 months.^10^ In our current study, the disease control rate for both cohorts combined was modest at 30%. The best clinical response was stable disease in the Durva/Treme + IR cohort (three patients) and partial response (1 patient) in the Durva/Treme cohort. Median PFS and OS were similar between both cohorts at 2–3 months and 5–6 months, respectively. These findings are comparable to previous reports; however, the study did not show any significant difference in efficacy between Durva/Treme + IR and Durva/Treme alone, and no patient was able to remain in the study for 6 months without progressing.

Molecular analysis of both baseline and on‐treatment biopsies showed a low TMB as well as minimal changes in immune cell composition posttreatment. As a known biomarker of response to immunotherapy, low TMB correctly predicted poor response in this population. However, given the small sample size and variable clinical response as well as known interindividual variability, identifying immune cell changes consistently in response to treatment or according to clinical results was unlikely in this small study.

For reasons that remain unclear, response to ICI therapy has not been as promising in BTC as it has in HCC. In our previous study, immune profiling from the samples of patients treated with tremelimumab and ablation revealed differences in the pattern of change of immune cell subsets between HCC and BTC cells.^10^ Although HCC and BTC both originate from the liver, the different paths of tumorigenesis, the distinctive tumor immune response of BTC versus HCC, and/or the low mutational burden of BTC may play a role.

The lack of efficacy difference between the two treatment arms may be due to the fact that the study population consisted of patients with refractory disease who had already received at least one line of systemic therapy. This may suggest that Durva/Treme combination therapy may be more effective in the first‐line setting when the tumor burden is lower and the immune system has not been as heavily compromised by prior therapy. This possibility is supported by a previous study that used Durva/Treme as first‐line therapy in patients with hepatocellular carcinoma and saw a median OS of ~16 months.^44^ Previous studies have also found that the addition of chemotherapy to ICI improves outcomes,^45^ and future studies could assess the combination of all three agents: ICI, chemotherapy, and IR. Other possible reasons for similar PFS and OS rates in both cohorts in this study may be related to the use of IR. Although IR is a commonly used approach for treating liver tumors, it may not be effective in all patients with BTC or may work better in some patients than in others. It is possible that patients in the Durva/Treme + IR arm had tumors that were not responsive to this type of treatment, which may have impacted the overall efficacy of the combination therapy.

Limitations of this study include the small sample size and the heterogeneous nature of BTC. Patients in this study had different baseline disease status and primary tumor locations. Future assessment of the combination treatment in large randomized studies should consider these factors.

## 5 CONCLUSION

Although we did not find any significant difference in efficacy measures between the two cohorts, the safety profile was manageable and similar for both cohorts. This finding could provide reassurance for clinicians who may consider combining ICIs with IR in patients with advanced BTC. Given the small size of the evaluable study population, the interpretation of results is limited. The combination of durvalumab and tremelimumab with or without IR is safe and larger studies are needed to fully evaluate efficacy.

## AUTHOR CONTRIBUTIONS

**Cecilia Monge:** Conceptualization (supporting); data curation (supporting); formal analysis (supporting); funding acquisition (supporting); investigation (equal); methodology (equal); project administration (supporting); resources (supporting); software (supporting); supervision (supporting); validation (supporting); visualization (supporting); writing – original draft (lead); writing – review and editing (equal). **Changqing Xie:** Conceptualization (equal); data curation (equal); formal analysis (equal); funding acquisition (supporting); investigation (equal); methodology (equal); project administration (equal); resources (supporting); software (supporting); supervision (supporting); validation (supporting); visualization (supporting); writing – original draft (supporting); writing – review and editing (equal). **Yuta Myojin:** Conceptualization (equal); data curation (lead); formal analysis (lead); funding acquisition (supporting); investigation (equal); methodology (supporting); project administration (supporting); resources (supporting); software (lead); supervision (supporting); validation (equal); visualization (lead); writing – original draft (supporting); writing – review and editing (equal). **Kelley L. Coffman D'Annibale:** Conceptualization (equal); data curation (equal); formal analysis (supporting); funding acquisition (supporting); investigation (equal); methodology (supporting); project administration (supporting); resources (supporting); software (supporting); supervision (supporting); validation (supporting); visualization (supporting); writing – original draft (supporting); writing – review and editing (equal). **Donna Hrones:** Conceptualization (equal); data curation (supporting); formal analysis (supporting); funding acquisition (supporting); investigation (equal); methodology (supporting); project administration (supporting); software (supporting); supervision (supporting); validation (supporting); visualization (supporting); writing – original draft (supporting); writing – review and editing (supporting). **Gagandeep Brar:** Conceptualization (supporting); data curation (supporting); formal analysis (supporting); funding acquisition (supporting); investigation (equal); methodology (supporting); project administration (supporting); resources (equal); software (supporting); supervision (supporting); validation (supporting); visualization (supporting); writing – original draft (supporting); writing – review and editing (supporting). **Sophie Wang:** Conceptualization (supporting); data curation (supporting); formal analysis (supporting); funding acquisition (supporting); investigation (supporting); methodology (supporting); project administration (equal); resources (supporting); software (supporting); supervision (supporting); validation (supporting); visualization (supporting); writing – original draft (supporting); writing – review and editing (supporting). **Anuradha Budhu:** Conceptualization (supporting); data curation (supporting); formal analysis (supporting); funding acquisition (supporting); investigation (supporting); methodology (supporting); project administration (supporting); resources (supporting); software (supporting); supervision (supporting); validation (supporting); visualization (supporting); writing – original draft (supporting); writing – review and editing (supporting). **William D. Figg:** Conceptualization (supporting); data curation (supporting); formal analysis (supporting); funding acquisition (supporting); investigation (equal); methodology (equal); project administration (supporting); resources (supporting); software (supporting); supervision (supporting); validation (supporting); visualization (supporting); writing – original draft (supporting); writing – review and editing (supporting). **Maggie Cam:** Conceptualization (supporting); data curation (lead); formal analysis (lead); funding acquisition (supporting); investigation (supporting); methodology (supporting); project administration (supporting); resources (supporting); software (lead); supervision (supporting); validation (supporting); visualization (lead); writing – original draft (supporting); writing – review and editing (supporting). **Richard Finney:** Conceptualization (supporting); data curation (lead); formal analysis (lead); funding acquisition (supporting); investigation (supporting); methodology (supporting); project administration (supporting); resources (supporting); software (lead); supervision (supporting); validation (supporting); visualization (lead); writing – original draft (supporting); writing – review and editing (supporting). **Elliot B. Levy:** Conceptualization (supporting); data curation (supporting); formal analysis (supporting); funding acquisition (supporting); investigation (equal); methodology (supporting); project administration (supporting); resources (supporting); software (supporting); supervision (supporting); validation (supporting); visualization (supporting); writing – original draft (supporting); writing – review and editing (supporting). **David Kleiner:** Conceptualization (equal); data curation (equal); formal analysis (equal); funding acquisition (equal); investigation (equal); methodology (equal); project administration (equal); resources (equal); software (equal); supervision (equal); validation (equal); visualization (equal); writing – original draft (equal); writing – review and editing (equal). **Seth Steinberg:** Conceptualization (supporting); data curation (lead); formal analysis (lead); funding acquisition (supporting); investigation (supporting); methodology (supporting); project administration (supporting); resources (supporting); software (lead); supervision (supporting); validation (lead); visualization (lead); writing – original draft (supporting); writing – review and editing (equal). **Xin Wei Wang:** Conceptualization (supporting); data curation (lead); formal analysis (supporting); funding acquisition (supporting); investigation (supporting); methodology (supporting); project administration (supporting); resources (supporting); software (supporting); supervision (supporting); validation (supporting); visualization (supporting); writing – original draft (supporting); writing – review and editing (supporting). **Bernadette Redd:** Conceptualization (supporting); data curation (supporting); formal analysis (supporting); funding acquisition (supporting); investigation (equal); methodology (equal); project administration (supporting); resources (equal); software (supporting); supervision (supporting); validation (supporting); visualization (supporting); writing – original draft (supporting); writing – review and editing (supporting). **Bradford J. Wood:** Conceptualization (equal); data curation (supporting); formal analysis (supporting); funding acquisition (supporting); investigation (equal); methodology (supporting); project administration (supporting); resources (supporting); software (supporting); supervision (supporting); validation (supporting); visualization (supporting); writing – original draft (supporting); writing – review and editing (supporting). **Tim Greten:** Conceptualization (lead); data curation (equal); formal analysis (equal); funding acquisition (lead); investigation (lead); methodology (lead); project administration (lead); resources (lead); software (equal); supervision (lead); validation (equal); visualization (equal); writing – original draft (equal); writing – review and editing (lead).

## FUNDING INFORMATION

T.F.G. is supported by the Intramural Research Program of the NIH, NCI (ZIA BC 011343). The study was supported by the Intramural Research Program of the NIH, National Cancer Institute, Center for Cancer Research and a Cooperative Research and Development Agreement between NCI and AstraZeneca. CM received a fellowship award from the Cholangiocarcinoma Foundation and is a recipient of the Robert A. Winn Diversity in Clinical Trials Career Development Award.

## CONFLICT OF INTEREST STATEMENT

These studies pose no conflicting financial interests for any of the authors.

## Supporting information


Figure S1.

## Data Availability

The data generated for this study are available from the corresponding author upon reasonable request.
