# Supplementary figures and images for: Combined immune checkpoint inhibition with durvalumab and tremelimumab with and without radiofrequency ablation in patients with advanced biliary tract carcinoma
Source: Cancer Med. 2024 Jan 11;13(3):e6912. doi: 10.1002/cam4.6912 (PMC10904979; doi:10.1002/cam4.6912)

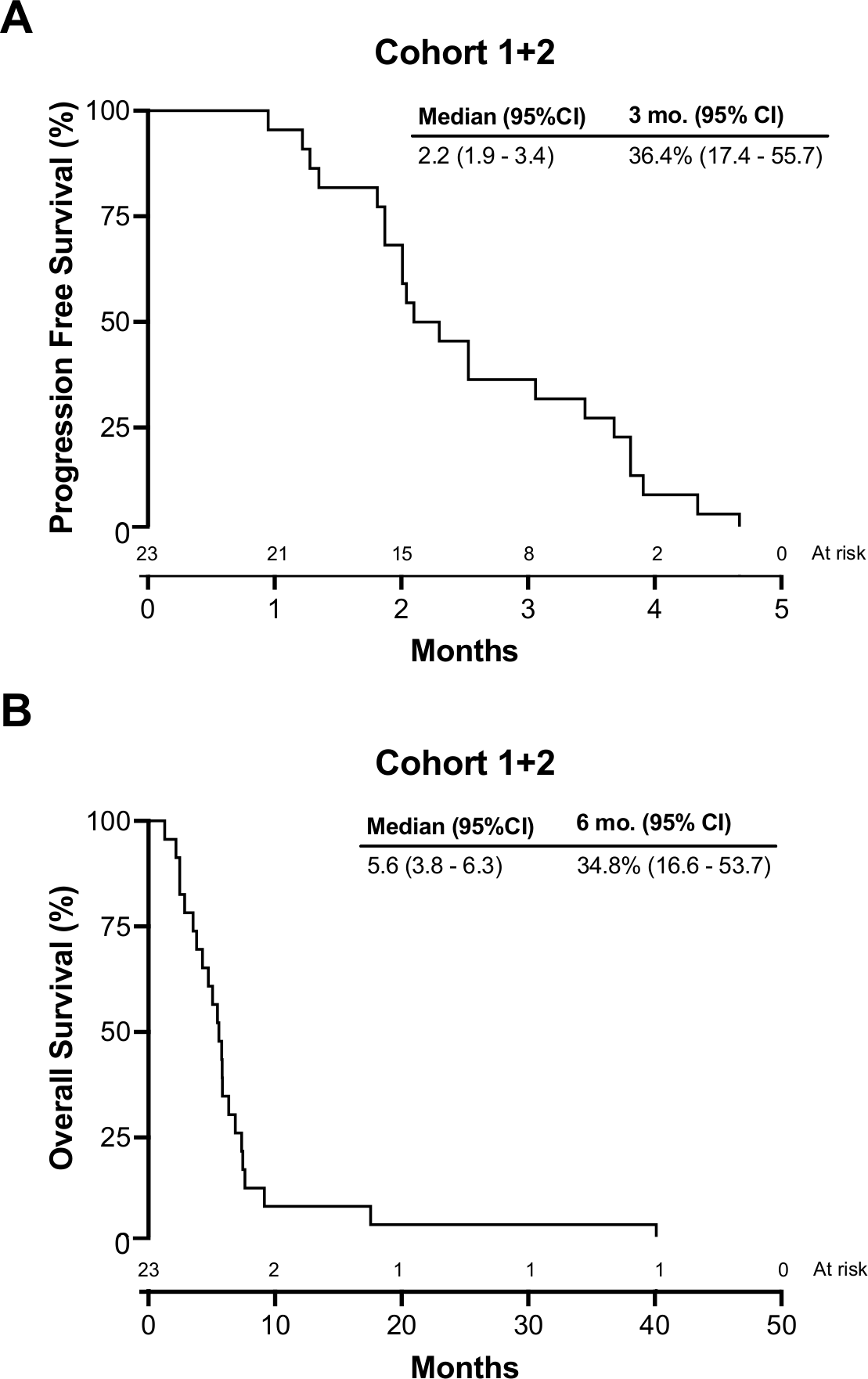

Supplement: Supplementary file 1 — Figure S1. [file CAM4-13-e6912-s001.tif]
